# Comparison of the therapeutic effects of open psychiatric wards for patients with depression: a meta-analysis

**DOI:** 10.3389/fpsyt.2026.1783980

**Published:** 2026-06-12

**Authors:** Pingan Ni, Peiling Yao, Kaili Zhu

**Affiliations:** Department of Psychosomatic Disorders, Huzhou Third People’s Hospital, Huzhou, China

**Keywords:** depressive disorder, meta-analysis, open-door policy, psychiatric department, hospital, treatment adherence

## Abstract

**Objective:**

To systematically evaluate and compare the differences in treatment outcomes between open and closed psychiatric wards for hospitalized patients with depression, and to provide evidence-based support for optimizing psychiatric ward management models.

**Methods:**

PubMed, Web of Science, China National Knowledge Infrastructure (CNKI), Wanfang Data, and VIP Database were searched for relevant literature published from inception to December 20, 2025. Randomized controlled trials (RCTs) comparing the therapeutic effects of open versus closed psychiatric wards in hospitalized patients with depression were included. The methodological quality of included studies was assessed using the Cochrane Risk of Bias tool. Meta-analysis was performed using RevMan 5.4.

**Results:**

A total of 9 RCTs were included. The meta-analysis showed that the open psychiatric ward group achieved better outcomes than the closed psychiatric ward group in depression symptom scores, with statistically significant differences in both the Self-Rating Depression Scale (SDS) score [MD = −3.81, 95% CI (−4.22, −3.40)] and the Hamilton Depression Rating Scale (HAMD) score [MD = −0.99, 95% CI (−1.18, −0.81)] (both P < 0.01). In terms of treatment adherence, the open ward group was higher than the closed psychiatric ward group [RR = 1.23, 95% CI (1.04, 1.47), P = 0.02]. There was no statistically significant difference between the two groups in clinical response rate [RR = 1.09, 95% CI (0.92, 1.29), P = 0.32]. Neither Egger’s regression test nor Begg’s rank correlation test indicated significant publication bias.

**Conclusion:**

Compared with closed psychiatric wards, open psychiatric wards may help improve depressive symptoms and increase treatment adherence among hospitalized patients with depression; however, their effect on clinical response rate remains unclear. Future high-quality, multicenter studies are needed to further verify the efficacy and safety of open psychiatric wards.

## Introduction

Depression is a common and severe mental disorder, with its high prevalence, recurrence, and disability rates posing a significant burden on individual functioning and public health (1, 2). While pharmacological and psychological treatments play important roles in managing depression, inpatient care remains a key therapeutic approach for patients with severe symptoms or significant suicide risk (3, 4). The management model of psychiatric wards, as an integral part of the inpatient treatment environment, may influence the treatment experience and clinical outcomes of patients with depression to some extent (5, 6). Traditional psychiatric inpatient treatment often employs closed or locked psychiatric ward management models, primarily aimed at reducing safety risks such as suicide, self−harm, and impulsive behaviors by restricting patients’ mobility (7, 8). However, while ensuring safety, closed psychiatric wards may also intensify patients’ feelings of passivity and loss of control, affecting their emotional experience and treatment cooperation (9, 10). In recent years, alongside shifts in mental healthcare philosophy emphasizing respect for patient rights and promoting recovery−oriented care, open psychiatric ward management models have gained increasing attention (11). Open psychiatric wards typically reduce restrictions on patients’ freedom of movement while maintaining basic safety, encouraging patients to actively engage in the treatment and rehabilitation process (8, 10, 12). It should be noted that there are certain differences in the management models of open and closed wards across different countries and regions. However, it is important to point out that the operational definitions of “open wards” vary significantly across different studies and national contexts. In the nine studies included in this review, the operational definition of an open ward was consistently “patients are free to leave and enter the ward during the daytime and return to the ward for rest at night”, with no mention of a completely unrestricted “fully open” policy, and necessary risk assessments and individualized restrictions were retained. For example, Nordic and some Western European countries (e.g., Norway, Germany) have adopted a more thorough open ward policy, emphasizing “openness” as the standard management model, with ward lockdowns occurring only in rare cases (8, 10). In contrast, most psychiatric wards in China that adopt “open management” still maintain certain access restrictions and supervision requirements, with considerable differences in ward structure, staffing, and the extent of openness (6, 7). Ward management models in different service contexts may have varying impacts on patient outcomes; therefore, the findings of this study should be interpreted with consideration of specific national contexts.

A key point of debate surrounding the open psychiatric ward management model is the balance between patient autonomy and institutional safety. Proponents argue that respecting patient autonomy and reducing coercive management measures help improve the therapeutic relationship and enhance patient motivation for treatment, aligning with the humanistic principles of modern mental health services (9). Opponents, however, express concern that open wards may increase safety risks such as patient self-harm, suicide, unauthorized leave, and impulsive behaviors, thereby posing legal and ethical challenges for healthcare institutions (8, 10). Differences in legal systems, allocation of medical liability, and mental health legislation across countries and regions also directly influence the implementation scope and regulatory details of open wards (8). Furthermore, cultural and legal factors affecting the implementation of open wards, as well as economic and cost-effectiveness considerations, cannot be overlooked. For instance, Nordic countries, due to higher nurse-to-patient ratios and well-established community mental health service systems, are able to implement open ward policies more broadly (8, 10). In contrast, regions with relatively limited resources may face more practical constraints in implementing open wards. These differences in background variables form the basis for comparisons across studies and represent important dimensions that this systematic review needs to address.

Existing studies suggest that open psychiatric wards may offer certain advantages in reducing aggressive behaviors, rates of restraint use, and improving patient satisfaction (9, 12, 13). However, current research has largely focused on safety or administrative indicators, and there is no consistent conclusion regarding whether open psychiatric wards can improve treatment outcomes in patients with depression, particularly key outcomes such as depressive symptom improvement and treatment adherence (7, 11−14). Furthermore, relevant studies exhibit considerable heterogeneity in research design, sample size, outcome measures, and definitions of ward management models, limiting the strength of evidence from individual studies and providing insufficient basis for clinical practice and management decisions. Currently, systematic reviews on the efficacy of open psychiatric wards in the inpatient treatment of depression remain scarce, particularly lacking quantitative synthesis of randomized controlled trial results. Therefore, it is necessary to comprehensively review and analyze existing evidence through systematic review and meta−analysis to more objectively evaluate the impact of open psychiatric wards on treatment outcomes in hospitalized patients with depression.

Based on this, this study systematically searched and included randomized controlled trials comparing the effects of open psychiatric wards versus closed (locked) psychiatric wards in hospitalized patients with depression. A meta−analysis was performed on outcome measures including depressive symptom scores, clinical response rate, and treatment adherence, aiming to provide evidence−based support for optimizing psychiatric ward management models and formulating inpatient treatment strategies for depression.

## Methods

### Study design

This study is a systematic review and meta−analysis designed to compare the treatment effects of open psychiatric wards versus closed (locked) psychiatric wards in hospitalized patients with depression. The research process followed the Preferred Reporting Items for Systematic Reviews and Meta−Analyses (PRISMA) guidelines (15). This study has not been registered on PROSPERO or any other platform. This statement is provided for clarification.

### Literature search strategy

A systematic computer-based search was conducted using the following databases: China National Knowledge Infrastructure (CNKI), Wanfang Database, VIP Database (VIP), PubMed, and Web of Science. The search timeframe spanned from the inception of each database to December 20, 2025. Specifically, the search coverage for CNKI, Wanfang Database, and VIP Database started from 1990, while the search coverage for PubMed and Web of Science started from 1980. The exact start dates for each database were determined based on their respective establishment dates and literature coverage scopes, in order to ensure comprehensiveness of the search. A combination of subject headings and free−text terms was used for the search. The English search terms included “depression”, “depressive disorder”, “open ward”, “open-door policy”, “closed psychiatric ward” and “locked psychiatric ward”. A retrospective search of the references in the included literature was conducted to supplement potentially omitted relevant studies.

### Literature inclusion and exclusion criteria

All nine articles included in this study are from China (6, 7, 11–14, 16–18). Therefore, the findings primarily reflect the efficacy of open psychiatric wards for hospitalized patients with depression in China. Caution is needed when generalizing these conclusions to other countries and regions (e.g., Europe, North America). Differences in mental health service systems, ward management models, and cultural backgrounds across countries may affect the generalizability of the results.

#### Inclusion criteria

(1) Subjects: Hospitalized patients with a clear diagnosis of depression or depressive disorder; (2) Intervention: Adoption of an open psychiatric ward management model or open-door policy; (3) Control: Adoption of a closed or locked psychiatric ward management model; (4) Outcome measures: Reporting at least one of the following outcome indicators: clinical response rate, Self-Rating Depression Scale (SDS), Hamilton Depression Rating Scale (HAMD), and treatment adherence; (5) Study types: Randomized controlled trials, cohort studies, or quasi-experimental studies; (6) Language of literature: Chinese or English. It should be noted that none of the included original studies fully reported baseline information such as the number of previous hospitalizations, duration of illness, or psychiatric and physical comorbidities. Therefore, the potential impact of these factors on treatment outcomes could not be controlled or assessed in the between-group comparisons.

#### Exclusion criteria

(1) Subjects comprising mixed psychiatric populations with data on depressive patients that could not be extracted separately; (2) Absence of a clear comparison between open and closed psychiatric ward management models; (3) No reported depression-related efficacy outcome indicators; (4) Reviews, systematic reviews, case reports, qualitative studies, theoretical articles, or conference abstracts; (5) Duplicate publications or literature with overlapping study subjects (the study with the larger sample size or most complete data was retained).

### Explanation regarding the composition of the ward team

It should be noted that none of the nine included original studies provided detailed information on the team composition in open versus closed wards, including the specific roles, number of staff allocated, and modes of involvement of psychiatrists, nurses, psychotherapists, occupational therapists, and other personnel. Potential differences in team composition across studies may have influenced treatment outcomes. However, due to the lack of relevant data in the original studies, this research was unable to conduct further analysis or intergroup comparisons in this regard, which constitutes one of the limitations of this study.

### Literature screening and data extraction

Two researchers independently screened the literature. First, titles and abstracts were reviewed to exclude studies that clearly did not meet the inclusion criteria, followed by full-text reading to determine the final included studies. A standardized data extraction form was used to extract information, including the first author, publication year, sample size, ward management model, outcome indicators, and measurement results. Discrepancies during the screening process were resolved through discussion and consensus. If disagreements persisted, a third researcher made the final decision.

### Definition of outcome indicators

The primary outcome measures included clinical response rate and depression symptom scores. The clinical response rate was defined by combining “remission”, “marked improvement”, and “response” based on the criteria for depression improvement in each study. It should be noted that the determination of “remission” in the included studies was based on the reduction rate of depression scale scores (e.g., HAMD or SDS), rather than on personal, social, or occupational functioning. Specifically, most studies adopted the following criteria: remission referred to a reduction rate of ≥75% on the HAMD or an SDS score falling within the normal range (<53 points); marked improvement referred to a reduction rate of 50%–74%; and response referred to a reduction rate of 25%–49%. Due to certain differences among studies in the use of scales and the thresholds for reduction rates, this meta-analysis used the original judgment results as reported in each study when pooling the data. Depression symptom scores were assessed using the SDS and HAMD. The secondary outcome measure was treatment adherence, typically expressed as the proportion of adherent patients during treatment relative to the total number of study participants.

### Risk of bias assessment

Randomized controlled trials were evaluated for quality using the Cochrane Risk of Bias Tool, covering random sequence generation, allocation concealment, blinding, completeness of outcome data, and selective reporting. Non-randomized studies were assessed using the Newcastle-Ottawa Scale (NOS). The risk of bias assessment was conducted independently by two researchers. Any discrepancies were resolved through discussion and consensus.

### Statistical analysis

Statistical analysis was performed using RevMan 5.4 software. Dichotomous outcome indicators (clinical response rate, treatment adherence) were expressed as relative risk (RR) with 95% confidence intervals (CI). Continuous outcome indicators (SDS, HAMD scores) were pooled for analysis using mean difference (MD) based on the consistency of the scales. Heterogeneity among studies was assessed using the χ² test and I² statistic. A fixed-effects model was applied when I² ≤ 50%, and a random-effects model was used when I² > 50%. Publication bias was quantitatively analyzed using Egger’s regression test and Begg’s rank correlation test. Statistical significance was set at P < 0.05.

## Results

### Literature screening results

**Figure 1:**
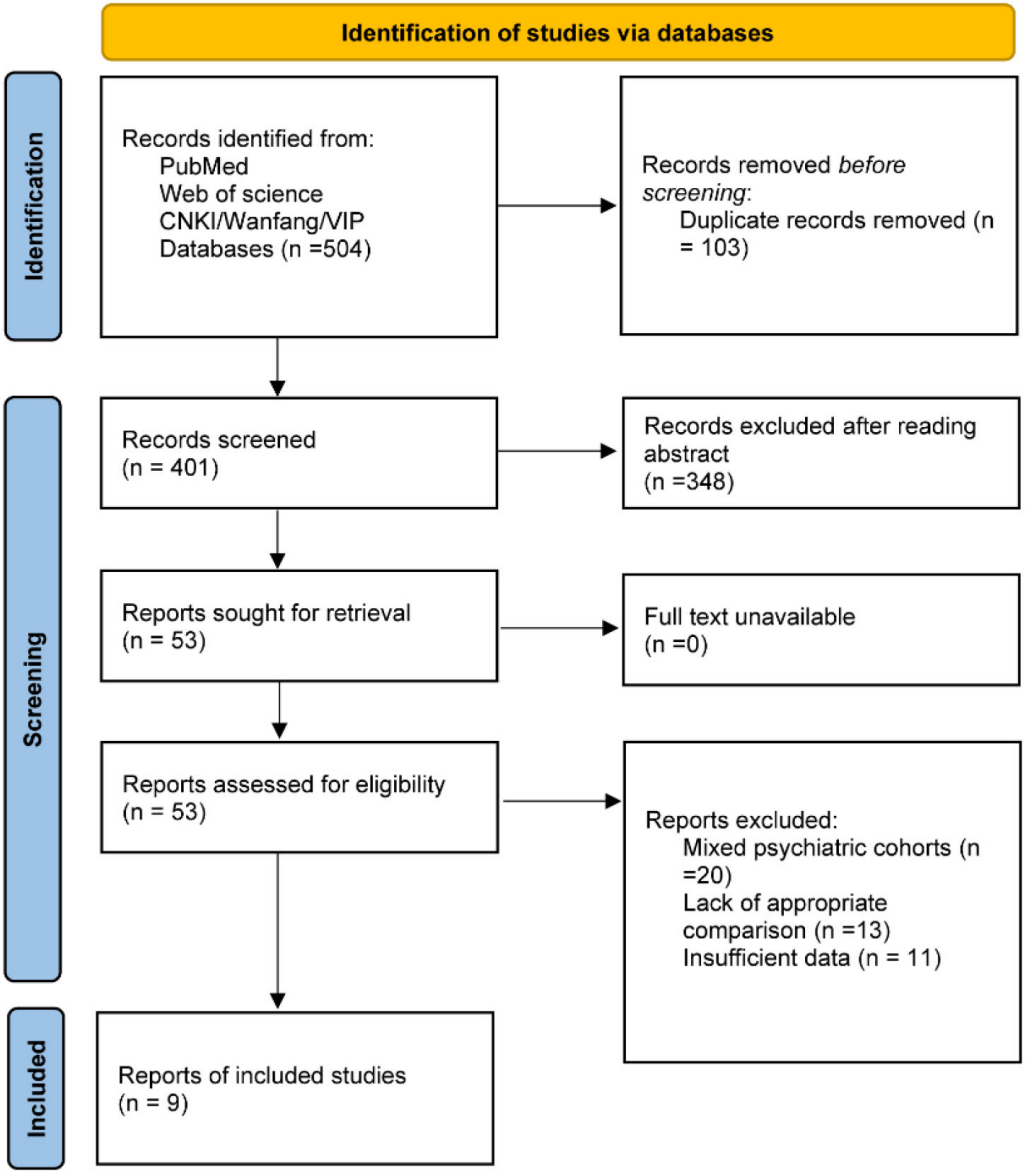
Flowchart of the literature screening process.

A systematic search of PubMed, Web of Science, CNKI, Wanfang Database, and VIP Database yielded 504 relevant articles. After removing 103 duplicates, 401 articles remained. Following an initial screening of titles and abstracts, 348 articles were excluded for not meeting the inclusion criteria, leaving 53 articles for full-text evaluation. After a detailed review of the full texts, 44 articles were excluded, including 20 with mixed psychiatric populations where data on depressive patients could not be separately extracted, 13 lacking appropriate comparisons between open and closed psychiatric ward models, and 11 with insufficient outcome data. Ultimately, 9 studies (6, 7, 11–14, 16–18) were included in the meta-analysis. The literature screening process is illustrated in **Figure 1**.

### Basic characteristics of included studies

A total of 9 studies were included in this research, all of which compared the therapeutic effects of open psychiatric wards versus closed psychiatric wards on hospitalized patients with depression. The publication years of the included studies ranged from 2010 to 2024, with a total sample size of 797 cases. In all studies, the experimental group adopted the open psychiatric ward management model, while the control group adopted the closed psychiatric ward management model. The general characteristics of the included studies are presented in **Table 1**.

**Table 1:** General information of included literature.

Author	Publication year	Sample size (n)		Age (years)		Gender ratio (male/female)		Intervention		Open ward operation mode	Closed ward operation mode	In-ward activities
		Experimental group	Control group	Experimental group	Control group	Experimental group	Control group	Experimental group	Control group			
Chen (6)	2018	46	46	23-42	23-42	16/30	16/30	Open Psychiatric Ward	Closed psychiatric Ward	open ward management, where patients are free to enter and exit the ward but must comply with ward regulations	closed management, where patient entry and exit are restricted and require permission from medical staff	health education, psychological counseling, and occupational and recreational activities
Gao (7)	2018	60	60	37-59	37-59	38/22	39/21	Open Psychiatric Ward	Closed psychiatric Ward	open ward management model encourages patients to participate in rehabilitation activities	routine closed ward management	rehabilitation training and psychological intervention
He (11)	2014	40	40	18-39	17-38	23/17	22/18	Open Psychiatric Ward	Closed psychiatric Ward	New open management model reduces restrictions and encourages autonomous participation	traditional closed management	psychological counseling and rehabilitation activities
He (12)	2024	32	33	24-56	25-55	17/15	18/15	Open Psychiatric Ward	Closed psychiatric Ward	Open nursing management allows patients free access	closed nursing management	psychological nursing and rehabilitation guidance
Luo (13)	2018	50	50	21-46	22-47	22/28	21/29	Open Psychiatric Ward	Closed psychiatric Ward	Open ward management in psychiatry	closed ward management in psychiatry	rehabilitation nursing and psychological support
Zhang (14)	2014	49	39	15-44	14-45	31/18	22/17	Open Psychiatric Ward	Closed psychiatric Ward	Open management model reduces restrictions	routine closed management	health education and psychological intervention
Zhang (16)	2015	55	53	22-46	22-46	N/A	N/A	Open Psychiatric Ward	Closed psychiatric Ward	Open management model	closed management model	maintenance treatment and rehabilitation guidance
Zhu (17)	2015	42	42	22-73	20-75	9/33	10/32	Open Psychiatric Ward	Closed psychiatric Ward	Open management and rehabilitation nursing	routine closed management	rehabilitation nursing and psychological support
Zhu (18)	2010	30	30	17-44	19-48	17/13	12/18	Open Psychiatric Ward	Closed psychiatric Ward	Open management	closed management	psychological intervention and rehabilitation activities

### Risk of bias assessment results

This study included a total of 9 randomized controlled trials (6, 7, 11–14, 16–18), all of which were evaluated for quality using the Cochrane Risk of Bias tool. All studies mentioned the method of randomization, and the risk of bias from random sequence generation was assessed as low. However, most studies did not provide detailed descriptions of allocation concealment methods, leading to an unclear risk of bias in this domain. Due to the nature of the interventions, blinding of participants and personnel was difficult to implement in the context of open psychiatric ward management; consequently, the risk of bias for this item was assessed as high across all studies. Reporting on blinding of outcome assessment was insufficient, with the risk mostly judged as unclear. All studies fully reported their outcome measures, with no significant instances of dropout or missing data identified. Therefore, the risks of bias from incomplete outcome data and selective reporting were both assessed as low. No other obvious sources of bias were found. The results are presented in **Table 2**. Overall, the methodological quality of the included studies was acceptable.

**Table 2:** Risk of bias assessment results for included studies.

Author	Publication year	Random sequence generation	Allocation concealment	Blinding (participants and personnel)	Blinding (outcome assessment)	Incomplete outcome data	Selective reporting	Other bias
Chen (6)	2018	Low risk	Unclear	High risk	Unclear	Low risk	Low risk	Low risk
Gao (7)	2018	Low risk	Unclear	High risk	Unclear	Low risk	Low risk	Low risk
He (11)	2014	Low risk	Unclear	High risk	Unclear	Low risk	Low risk	Low risk
He (12)	2024	Low risk	Unclear	High risk	Unclear	Low risk	Low risk	Low risk
Luo (13)	2018	Low risk	Unclear	High risk	Unclear	Low risk	Low risk	Low risk
Zhang (14)	2014	Low risk	Unclear	High risk	Unclear	Low risk	Low risk	Low risk
Zhang (16)	2015	Low risk	Unclear	High risk	Unclear	Low risk	Low risk	Low risk
Zhu (17)	2015	Low risk	Unclear	High risk	Unclear	Low risk	Low risk	Low risk
Zhu (18)	2010	Low risk	Unclear	High risk	Unclear	Low risk	Low risk	Low risk

### Meta-analysis results

#### Clinical effectiveness rate

**Figure 2:**
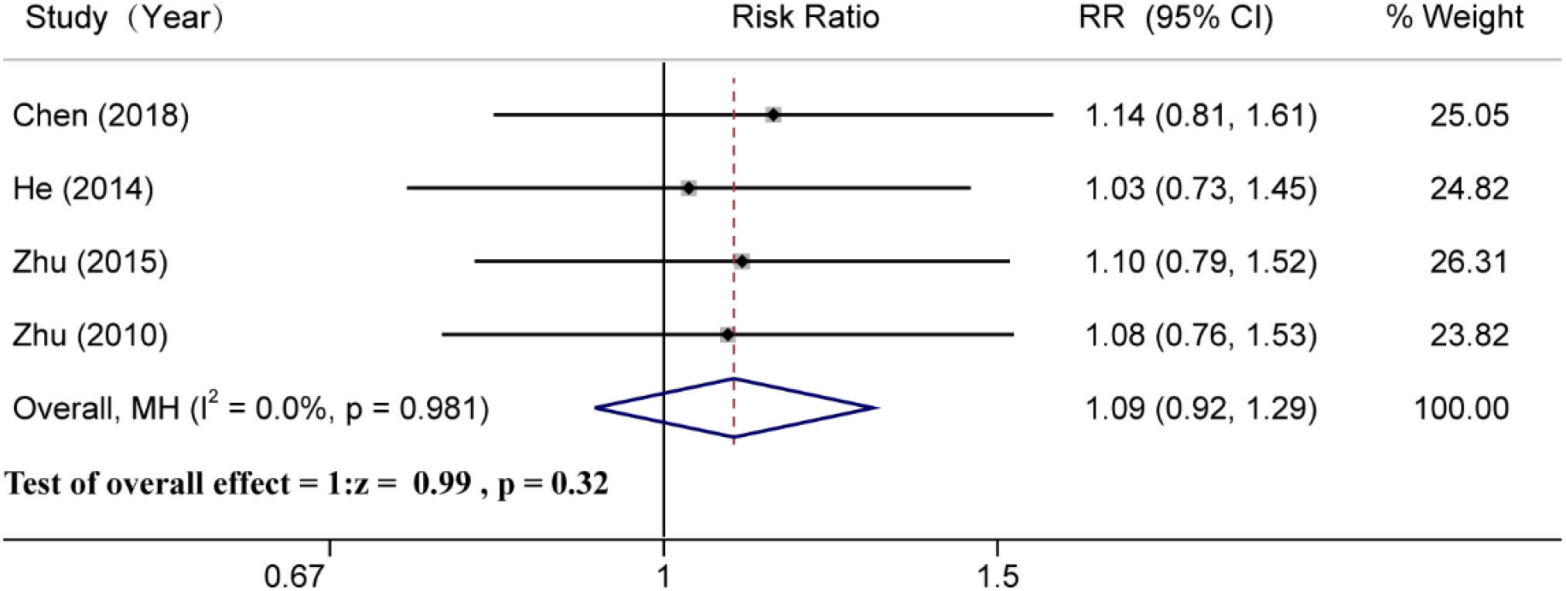
Forest plot of the meta-analysis comparing clinical effectiveness rates between open and locked psychiatric wards for patients with depression.

A total of four studies (6, 12, 17, 18) reported comparisons of the clinical effectiveness rate between open and locked psychiatric wards for patients with depression. Heterogeneity tests indicated no significant heterogeneity across studies (I² = 0.0%, P = 0.981); therefore, a fixed-effect model was applied for pooled analysis. The meta-analysis showed no statistically significant difference in the clinical effectiveness rate between the open psychiatric ward group and the locked psychiatric ward group [RR = 1.09, 95% CI (0.92, 1.29), P = 0.32], as shown in **Figure 2**.

#### SDS depression score

**Figure 3:**
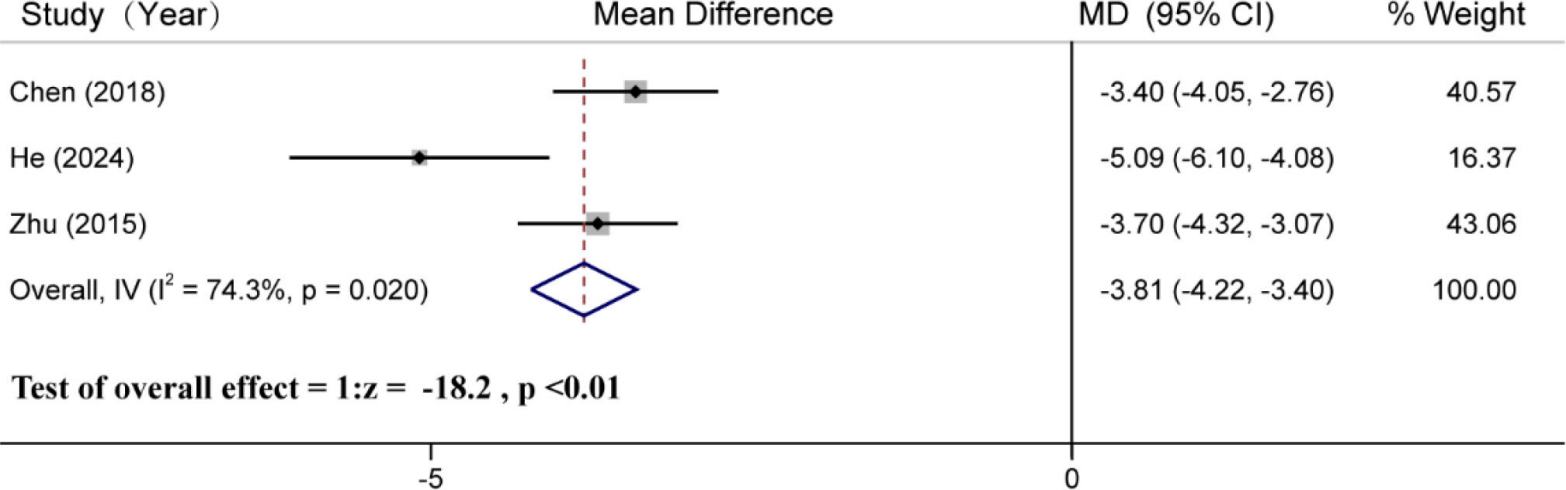
Forest plot of the meta-analysis comparing SDS depression scores in patients with depression between open and closed psychiatric wards.

Three studies (6, 12, 17) reported changes in SDS depression scores. Heterogeneity tests revealed substantial heterogeneity across studies (I² = 74.3%, P = 0.020), and a random-effects model was used for pooled analysis. The meta-analysis results indicated that the SDS depression score after treatment was significantly lower in the open psychiatric ward group compared to the locked psychiatric ward group [MD = −3.81, 95% CI (−4.22, −3.40), P < 0.01], as shown in **Figure 3**.

#### HAMD depression score

**Figure 4:**
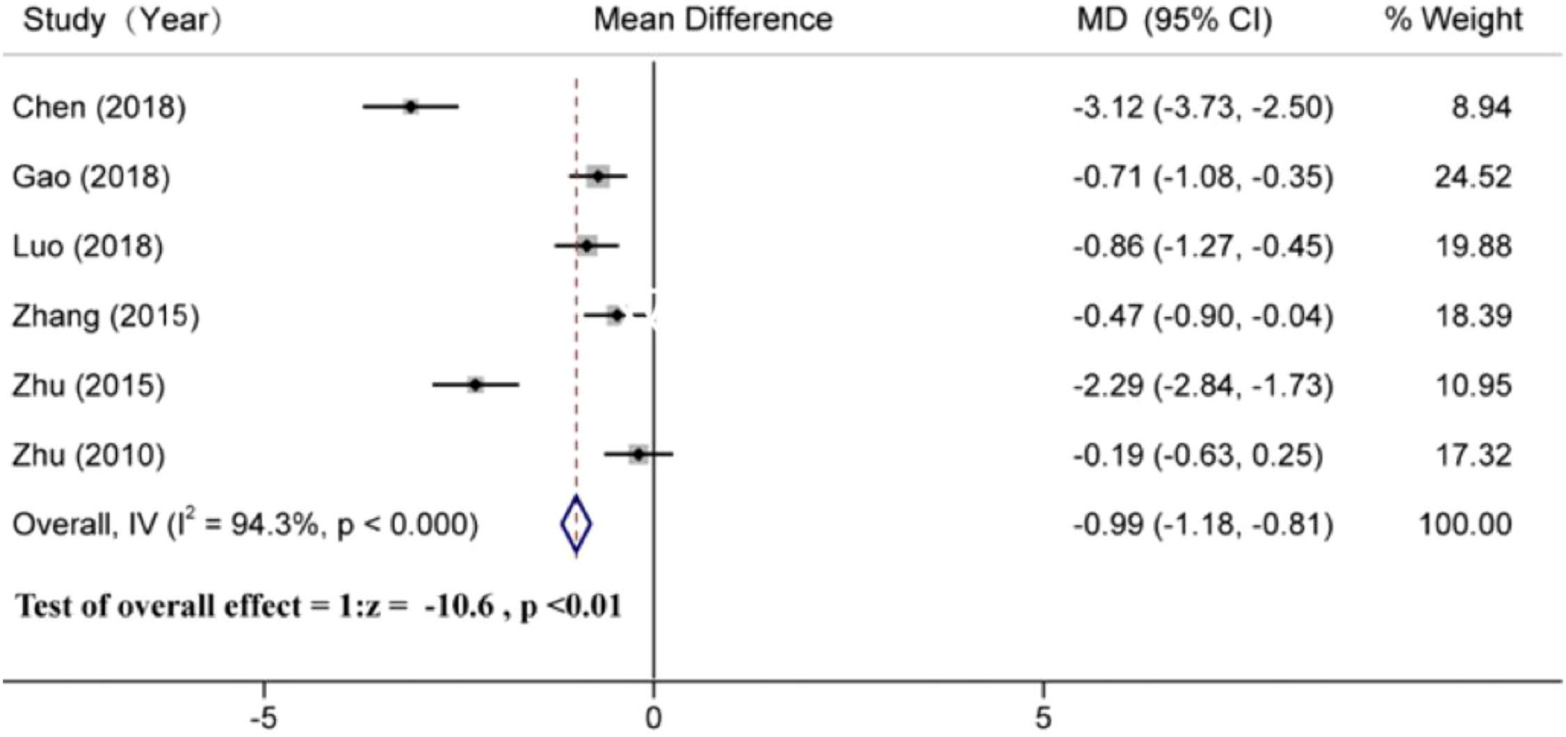
Forest plot of the meta-analysis comparing HAMD scores between open and locked psychiatric wards for patients with depression.

Six studies (6, 7, 13, 16–18) reported changes in HAMD depression scores. Heterogeneity tests showed considerable heterogeneity across studies (I² = 94.3%, P < 0.01), and a random-effects model was applied for pooled analysis. The meta-analysis demonstrated that the HAMD depression score after treatment was significantly lower in the open psychiatric ward group than in the locked psychiatric ward group [MD = −0.99, 95% CI (−1.18, −0.81), P < 0.01], as shown in **Figure 4**.

### Treatment adherence

**Figure 5:**
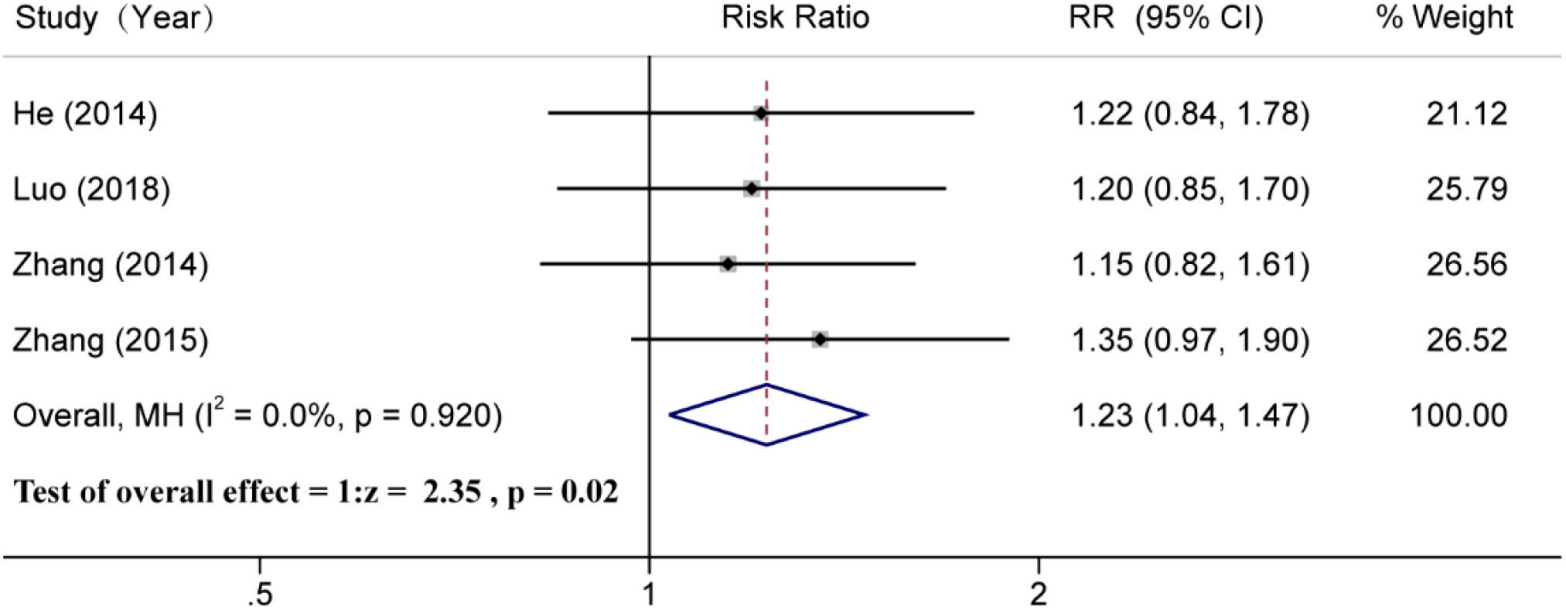
Forest plot of the meta-analysis comparing the treatment compliance of depression patients in open versus closed psychiatric wards.

Four studies (11, 13, 14, 16) reported treatment adherence. Heterogeneity tests indicated no significant heterogeneity across studies (I² = 0.0%, P = 0.920), and a fixed-effect model was used for pooled analysis. The meta-analysis revealed that treatment adherence was significantly higher in the open psychiatric ward group compared to the locked psychiatric ward group [RR = 1.23, 95% CI (1.04, 1.47), P = 0.02], as shown in **Figure 5**.

### Sensitivity analysis

Sensitivity analysis was conducted by excluding individual studies one at a time. The results showed that the pooled effect sizes for all outcome indicators did not change significantly, indicating good stability of the study findings.

### Publication bias analysis

Quantitative analysis of publication bias was performed using Egger’s regression test and Begg’s rank correlation test. The results indicated that neither Egger’s regression test (P = 0.45) nor Begg’s rank correlation test (P = 0.62) suggested significant publication bias.

## Discussion

This study employed a systematic review and meta-analysis to comprehensively evaluate the treatment outcomes of depressed inpatients in open versus closed psychiatric wards. The results indicated that open psychiatric wards have certain advantages in improving depression symptom scores (SDS and HAMD) and enhancing treatment adherence, whereas the difference in clinical response rates between the two ward management models did not reach statistical significance. Although this finding appears somewhat contradictory to the improvement observed in depression symptom scores, it can be explained from the following perspectives. First, clinical response rate is typically determined based on categorical reductions in scale scores (e.g., “remission,” “marked improvement,” “improvement”), and pooling such data across studies may reduce sensitivity due to inconsistencies in the criteria used to define response. In contrast, SDS and HAMD, as continuous variables, can more precisely reflect the magnitude of change in depressive symptoms and may therefore offer greater statistical power to detect differences between ward management models. Second, clinical response rate focuses on achieving a “threshold” level of symptom improvement, whereas the benefits of open wards may be more evident in the overall reduction of symptom severity rather than a substantial increase in the response rate. Taken together, open psychiatric wards may play a positive role in improving patients’ symptomatic experience and treatment-related outcomes, though their impact on traditional binary outcome measures requires further investigation. The aim of this study is to provide evidence-based support for optimizing psychiatric ward management models. These findings suggest that future research should not only focus on clinical response rates but also emphasize the use of continuous scale-based outcomes to more comprehensively assess the nuanced effects of management models on patient symptom improvement. The meta-analysis of depression symptom scores revealed that the open psychiatric ward management model significantly reduced SDS and HAMD scores. Compared with closed wards, open psychiatric wards emphasize a patient-centered management philosophy, reducing restrictions on patients’ freedom and autonomy imposed by the hospitalization environment (10). Previous studies have indicated that depression patients in closed or highly controlled inpatient settings are prone to experiencing feelings of passivity, helplessness, and stigma, which may diminish therapeutic effects to some extent (9, 19). A relatively open ward environment may help alleviate patients’ tension and sense of oppression, improving their subjective emotional experience and thereby promoting the remission of depressive symptoms (20). Furthermore, open psychiatric wards often facilitate improved doctor-patient interaction patterns. A more relaxed management environment may increase the frequency and quality of communication between healthcare providers and patients, making patients more willing to express their emotional states and treatment needs, thus strengthening the therapeutic alliance (21). A strong therapeutic alliance is considered one of the important predictors of treatment efficacy for depression, which may partly explain the advantage observed for open psychiatric wards in depression symptom scores (9, 22).

From a clinical and ethical perspective of critical reflection, the core value of the open ward model lies in its respect for patient autonomy. Traditional closed wards prioritize “safety first”, but this may come at the cost of patients’ self-determination and weaken their intrinsic therapeutic motivation. By granting patients greater freedom of movement and opportunities to participate in decision-making, open wards help restore their identity as active agents in treatment, which aligns closely with the “recovery-oriented” and “person-centered” principles advocated in contemporary mental health services (8, 10). However, while enhancing autonomy, open wards also introduce ethical tensions concerning safety. The open wards included in this study all retained necessary risk assessments and individualized restrictive measures, yet clinical practice still faces the challenge of delineating a reasonable boundary between respecting autonomy and ensuring safety. For example, in patients with major depressive disorder who present with severe suicide risk or impulse control disorders, excessive emphasis on openness may increase the risk of adverse events, whereas excessive restriction may violate the principle of patient autonomy (9, 22). The results of this study show that open wards have advantages in improving depressive symptoms and adherence, but safety outcomes (e.g., suicide, self-harm, unauthorized leave) were not included, so no definitive conclusions can be drawn regarding their safety. Improvement in the therapeutic alliance may serve as a key mediating mechanism linking autonomy and efficacy—when patients feel respected and trusted during treatment, their identification with the treatment plan and willingness to cooperate increase, thereby forming a virtuous cycle. In summary, the implementation of open wards is not a simple binary choice of “open versus closed”, but rather a dynamic decision-making process based on individualized patient risk assessment, ward resource allocation, and ethical deliberation.

This study found that treatment adherence was significantly better in the open psychiatric ward group compared to the closed ward group, with low heterogeneity among studies, suggesting good stability of this result. Treatment adherence is a critical factor influencing the efficacy of depression treatment; insufficient compliance can lead to poor pharmacological outcomes and increased risk of relapse. By reducing coercive management measures, open psychiatric wards may provide patients with a greater sense of participation and control during treatment, potentially enhancing their understanding and acceptance of the treatment regimen, thereby improving compliance (7, 11). This finding holds both statistical significance and clear clinical relevance. It is noteworthy that this study did not observe a significant advantage for open psychiatric wards in terms of clinical response rate. Clinical response rate is typically determined by grading symptom improvement and is influenced by assessment criteria and researchers’ subjective factors, potentially making it less sensitive than continuous scale indicators. Additionally, varying definitions of “effective” across studies may reduce the discriminative power of this indicator in meta-analysis. In contrast, scales such as SDS and HAMD can more finely reflect the magnitude of changes in depressive symptoms and may thus offer greater advantages in evaluating differences between ward management models (6, 11).

A certain degree of heterogeneity was observed in the meta-analyses of SDS and HAMD scores (6, 12, 17), which may be attributed to differences among the included studies in sample size, intervention duration, specific implementation of ward openness, and baseline severity of depression in patients. Despite the heterogeneity, the direction of the pooled effect sizes across studies was consistent, and the overall results were statistically significant, suggesting that the potential benefits of open psychiatric wards in improving depressive symptoms are relatively stable.

This study has several limitations. First, the number of included studies is relatively limited, and most are single-center studies. All included studies were conducted in China, and the findings primarily reflect the actual situation of psychiatric ward management models in China. Caution is needed when extrapolating the results to other countries or regions (e.g., Europe, North America), as there are significant differences in policy context, team composition, and management details of open wards across different countries and regions. Second, none of the included original studies fully reported key baseline information such as the number of previous hospitalizations, illness duration, and psychiatric or physical comorbidities, which limits the assessment of between-group comparability and may introduce potential confounding bias. Third, none of the included studies reported in detail the team composition (e.g., the specific roles and configuration of psychiatrists, nurses, psychotherapists, and occupational therapists) of open versus closed wards. Differences in team composition may directly affect intervention implementation outcomes, representing an important methodological limitation of this study. Fourth, some studies had insufficient reporting on allocation concealment and blinding procedures, which may introduce methodological bias. Fifth, inconsistencies in the description of specific implementation details of open psychiatric ward management models across studies increased heterogeneity in interventions. Finally, this study was not registered with the PROSPERO platform. Although this did not affect the rigor of the analysis process, it may have a certain impact on study transparency. Publication bias analysis was constrained by the limited number of included studies, and the results should be interpreted with caution.

In summary, the findings of this study suggest that, while ensuring inpatient safety management, open psychiatric wards may help improve depressive symptoms and enhance treatment adherence in hospitalized depression patients. However, their impact on traditional outcome measures such as clinical response rate remains unclear. Future multicenter, large-sample, high-quality randomized controlled trials are needed to further validate the long-term efficacy and safety of open psychiatric wards in the inpatient treatment of depression, providing more robust evidence-based support for optimizing psychiatric ward management models.

From the perspective of institutional decision-making, the findings of this study offer direct practical implications. For psychiatric institutions considering reforms to ward management models, the results suggest that open wards may be superior to traditional closed wards in improving patients’ depressive symptoms and treatment adherence, providing evidence-based support for the management philosophy of “prioritizing openness, supplemented by restrictions”. When implementing open wards, healthcare institutions should simultaneously establish robust risk assessment systems and individualized management protocols to balance the tension between patient autonomy and institutional safety. Furthermore, decision-makers need to tailor the implementation details of open wards to local conditions, taking into account regional healthcare resource allocation, the legal environment, and patient population characteristics, rather than simply applying the study conclusions uncritically. The efficacy data from this study provide an evidence-based foundation for optimizing management models; however, careful clinical judgment is still required during the translation of these findings into practice.

## Data Availability

The original contributions presented in the study are included in the article/supplementary material, further inquiries can be directed to the corresponding author/s.
